# Differential Screening of Phage-Ab Libraries by Oligonucleotide Microarray Technology

**DOI:** 10.1371/journal.pone.0001508

**Published:** 2008-01-30

**Authors:** Paolo Monaci, Alessandra Luzzago, Claudia Santini, Alessandra De Pra, Mirko Arcuri, Francesca Magistri, Alessandro Bellini, Helenia Ansuini, Maria Ambrosio, Virginia Ammendola, Maria Giulia Bigotti, Agostino Cirillo, Maurizio Nuzzo, Annamaria Assunta Nasti, Philippe Neuner, Laura Orsatti, Monica Pezzanera, Andrea Sbardellati, Giuseppe Silvestre, Paolo Uva, Valentina Viti, Gaetano Barbato, Stefano Colloca, Anna Demartis, Emanuele De Rinaldis, Saverio Giampaoli, Armin Lahm, Fabio Palombo, Fabio Talamo, Alessandra Vitelli, Alfredo Nicosia, Riccardo Cortese

**Affiliations:** 1 Biotechnology Department, Istituto di Ricerca di Biologia Molecolare (IRBM) P. Angeletti, Pomezia, Rome, Italy; 2 Centro di Ricerca per l'Ingegneria Genetica (CEINGE), Napoli, Italy; Texas Tech University Health Sciences Center, United States of America

## Abstract

A novel and efficient *tagArray* technology was developed that allows rapid identification of antibodies which bind to receptors with a specific expression profile, in the absence of biological information. This method is based on the cloning of a specific, short nucleotide sequence (*tag*) in the phagemid coding for each phage-displayed antibody fragment (phage-Ab) present in a library. In order to set up and validate the method we identified about 10,000 different phage-Abs binding to receptors expressed in their native form on the cell surface (10 k *Membranome* collection) and tagged each individual phage-Ab. The frequency of each phage-Ab in a given population can at this point be inferred by measuring the frequency of its associated tag sequence through standard DNA hybridization methods. Using tiny amounts of biological samples we identified phage-Abs binding to receptors preferentially expressed on primary tumor cells rather than on cells obtained from matched normal tissues. These antibodies inhibited cell proliferation *in vitro* and tumor development *in vivo*, thus representing therapeutic lead candidates.

## Introduction

In recent years monoclonal antibodies (mAbs) have proven to be excellent therapeutic agents [1], [2]. They have long half-life, favorable pharmaco-kinetics in humans, none or very few adverse reactions and a well established industry-scale production process [1], [3], [4]. But their most significant feature is the ability to specifically bind very diverse molecules with high affinity. Among these, the receptors expressed on the surface of the eukaryotic cell are particularly important as therapeutic targets. These receptors mediate the response of the cell to environmental stimuli, and thus it is not surprising that they play a key role in a large number of diseases, including cancers, infections and auto-immune diseases (e.g. Crohn's disease, rheumatoid arthritis, asthma).

Therapeutic mAbs are generally selected following the identification of a suitable target. Often, key information is provided by large scale transcriptional studies which identify genes differentially expressed in cells under normal and pathological conditions. However this approach omits several potentially interesting targets, such as those whose protein expression level does not vary in tandem with the corresponding mRNA level, or those whose structure/epitope is dependant on a post-translational protein modification, or even those cases in which protein-protein interactions generate novel, pathologically related epitopes. We therefore devised a highly sensitive method to survey the differential binding of a large number of clones which can be adapted to the very small scale of tissues biopsies. This strategy allows the identification of epitopes with a specific expression profile (e.g., tumor-specific), independently of any biological information. The method is based on i) the availability of a defined collection of phage-Abs binding to the epitopes of membrane proteins (i.e. the *Membranome* collection) and ii) the possibility of tagging every phage-Ab with a specific DNA tag sequence.

*In silico* analysis of the human genome predicts the total size of the membrane protein (MP) coding genes-the *Membranome*–to be less than 5,000. Thus, by using high throughput screening methods and phage-displayed libraries, it is feasible to put together a collection containing antibodies that bind to all or most MPs. Here we report the creation of just such a large and diverse collection of Ab fragments binding MPs, generated by panning phage-displayed libraries of single-chain antibodies (scFv) on whole cells. Furthermore, we inserted a unique oligonucleotide tag sequence in each phagemid of the *Membranome* collection, and generated two populations by panning the tagged collection on tumor and on normal tissue. Tumor-specific phage-Abs were identified by comparing the frequency of each tag between the two populations. This was quickly achieved by hybridizing the tag populations to a customized tag-array microchip. The tumor-specific phage-Abs can be used to identify their target and, after conversion into human IgGs, for the phenotypic analysis of their biological properties.

## Results

### Strategy for generating a collection of phage-Ab binding *Membranome* proteins

We define as *Membranome* the ensemble of human genes coding for proteins associated to the cell membrane. *In silico* analysis identified about 4,600 genes populating the human *Membranome* (approx 20% of the human genome; see Materials and methods). We built up a collection of phage-Ab that bind to the *Membranome* proteins by high-throughput *in vitro* screening of naïve phage-Ab libraries, using various cell lines as “selectors”. MPs expressed on the cell surface maintain their native features: folding, post-translation modification, expression of splicing variants and formation of multimeric complexes. In addition, cultured cell lines are reagents readily available with reproducible properties. We analyzed *in silico* the variation in expression levels of MP-encoding genes in a set of 25 cell lines (see Table S1). For each cell line we ranked the MP-encoding genes according to their level of gene expression. We considered genes expressed in a given cell line when included in the top ranking expression level of 200, 300 or 400. This analysis reveals that about 30% of the MP-encoding genes are expressed in only one of the 25 cell lines, whereas only a small fraction (around 2–3%) are expressed in all the cell lines. These data provide an estimate of the diversity of expression of the MP- encoding genes across different cell lines. They also indicate that increasing the number of cell lines generates a set expressing as a whole an increasing number and eventually all the MP-encoding genes.

### Building up the *Membranome* phage-Ab repository

**Figure 1:**
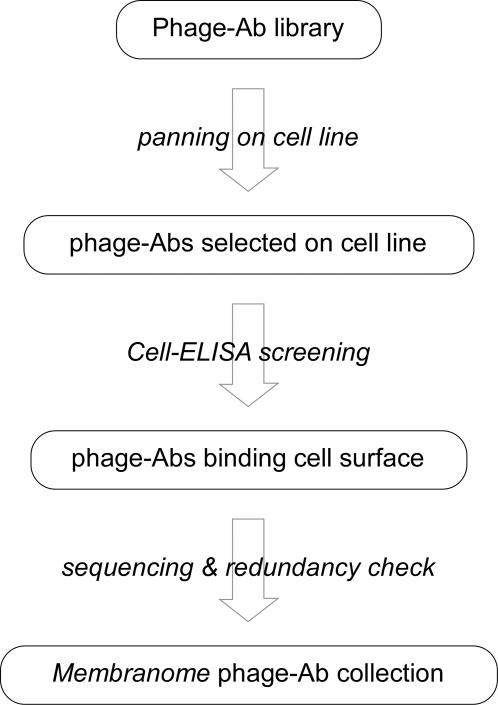
Strategy adopted to generate the 10k *Membranome* phage-Ab collection

The workflow adopted to build up the *Membranome* phage-Ab collection is schematically described in Figure 1. We used three naïve phage-Ab libraries derived from B cells of human healthy donors which include over 10^10^ different clones each. [5]. These libraries were panned separately on 64 different human cell lines derived from 25 different tissues (see Table S2). We performed only two rounds of panning to preserve the complexity of the selected population of phage and limit the preferential amplification of the most biologically viable clones. Phage-Abs from each selected pool were individually screened by cell-ELISA for their ability to bind the selector cell line. Positive clones with a novel VH-CDR3 sequence were progressively added to the *Membranome* phage-Ab collection. Automation of this process enabled high throughput screening, and more than 112,000 clones were analyzed by cell ELISA. Among these, about 40,000 clones (41%) were confirmed positive and found to include 9,925 different VH-CDR3 sequences (8.8%). This set of 9,925 clones is referred to as the 10k *Membranome* phage-Ab collection. On average, 290 unique phage clones were selected from each cell line and over 53% of these had a new VH-CD3 sequence. The frequency (and also the number) of the new phage-Abs clones derived from each cell line is steady, and not affected by the order in which each cell line was used for selection. This strongly suggests that this process, in addition to efficiently selecting a core of antibodies that recognize common surface proteins, also selects antibodies against target antigens over-expressed in a given cell line, thus enhancing the repertoire of binding specificities included in the collection.

### Use of the *Membranome* phage-Ab collection

The 10 k *Membranome* phage-Ab collection is a “specialized”, low-complexity library of clones which bind receptors expressed in their native form on the cell surface. This collection can be surveyed to rapidly identify antibodies binding epitopes of known cell-surface receptors. The most attractive use of the *Membranome* collection, however, is to select antibodies targeting epitopes of unknown receptors with specific expression features, which is the case of the tumor-associated receptors specifically over-expressed in tumor. Identifying such antibodies, however, is a goal barred by the lack of an efficient protocol for differential screening. Let us consider the case of a target and a reference biological sample. Panning the *Membranome* phage-Ab collection on each of these samples generated two phage populations. The goal is to identify clones preferentially or exclusively present in one of the two populations. Testing the binding specificity of individual clones from these populations would require large quantities of biological samples, which generally are available only in limited quantities, and would survey only a minor part of the population.

We addressed the problem from a different perspective. Sequencing a number of clones from the test and the reference population of phage-Ab could reveal clones which are differentially represented in the two pools, but clearly, a statistically significant comparison of the two populations would require a very large number of sequences. To efficiently acquire this information we developed a novel DNA-based screening technology we called *tagArray*. First we designed a set of short oligonucleotides that could be used as tags and cloned each single tag into each phagemid coding for a phage-Ab (see Materials and methods for details). In this way we generated an association between a specific tag and a specific scFv sequence, which makes it possible to measure the frequency of each scFv clone in a population by measuring frequency of the associated tag. Moreover, standard phage-binding screening protocols can be replaced by well-established DNA amplification and hybridization methods and high-throughput technologies, such as DNA microarray. The tagArray technology enables the entire populations to be compared in an efficient and sensitive way, using minimal amounts of selector samples. The screening protocol is organized in four steps: i) the tagged phage population is panned in parallel on a biological and a reference sample generating two distinct phage populations; ii) tag sequences are amplified from the target and reference phage populations, labeled with different fluorochromes and hybridized on a double channel DNA microarray containing the complete repertoire of tag sequences; iii) analysis of the hybridization data identifies the tags differentially represented in the two populations; iv) rescue of the scFv sequence associated to the relative tags are rescued from the phage population (see Materials and methods).

### Identification of phage-Abs that bind tumor cells by *tagArray* screening

**Figure 2:**
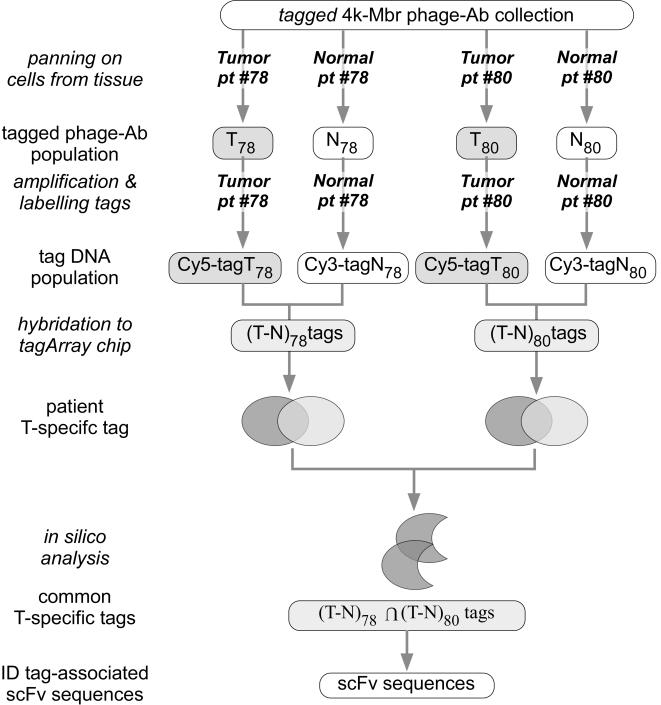
Workflow of the *tagArray* screening procedure employed to identify tumor-specific phage-Abs.

During the process of collecting the 10,000 phage-Ab of the 10 k-Membranome collection, we generated a preliminary version composed of the first 4,000 clones that were identified (4k-Membranome phage-Ab collection). A set of tag sequences was inserted in the phagemids of the 4k-*Membranome* collection, as detailed in Materials and methods. Biopsy specimens from tumor and adjacent normal tissues were collected from two colorectal cancer (CRC) patients (pt#78 and pt#80), samples were disaggregated by enzymatic treatment and the epithelial cells affinity purified. The tagged 4 k *Membranome* collection was panned on these primary epithelial cells to generate two pairs of tumor (T) and normal (N) phage populations (T_78_ and N_78_; T_80_ and N_80_; Figure 2). Tag sequences from each T and N matched population were amplified, labeled with different fluorochromes, mixed and hybridized to a DNA microarray containing the complete repertoire of tag sequences. The intensity of the two fluorochromes was measured for each spot of the microarray and analyzed as average intensity and ratio from two independent fluor-reversal experiments. The former reflects the average abundance whereas the latter reveals the differential frequency of each tag in the matched populations. Candidate tags were selected on the basis of statistical criteria (see Materials and methods) limiting our analysis to tags whose average intensity was higher than log_2_10. Since we aimed at identifying clones specifically enriched in the T as compared to the matched N phage population, we focused our attention on tags exhibiting the highest T/N ratio in both patients (average T/N ratio higher than 70% of the maximal ratio detected at the same intensity). This process identified a set of 174 tags among which we randomly chose a subset of 61 elements. By utilizing a nested PCR amplification protocol employing primers encompassing the tag sequence, we recovered 45 different phage-Abs associated with the selected tags from the T populations. This partial redundancy in the phage-Abs identified was expected since, under the experimental conditions adopted, a phage-Ab can be associated to more than one tag.

**Figure 3:**
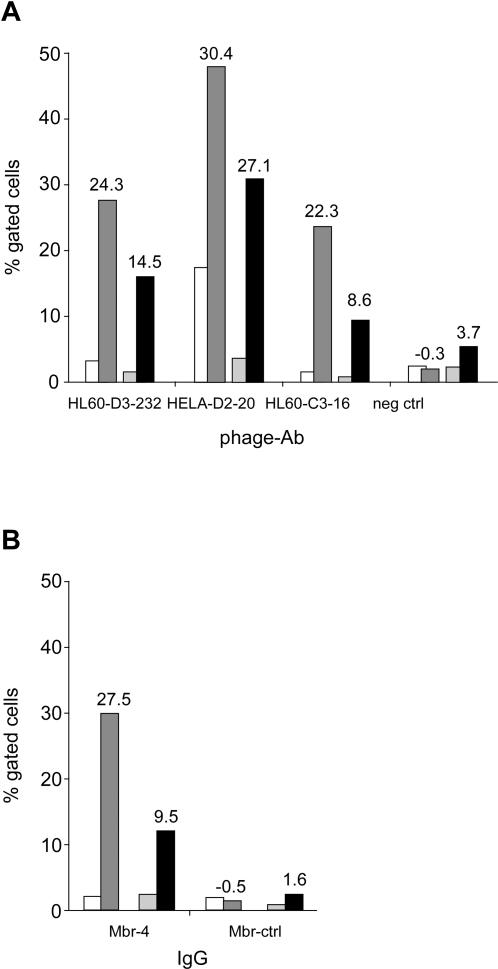
Binding specificity of selected clones. Binding of phage-Ab HL60-D3-232, HeLa-D2-20, HL60-C3-16 and a control phage-Ab (a) or of Mbr-4 and a control isotypic IgG (Mbr-ctrl; b) to human epithelial primary cells was measured by flow cytometry. Primary cells were obtained from matched tumor and adjacent normal tissue from CRC patients #78 (white and dark grey bars, respectively) and #80 (pale grey and black bars, respectively). Data are reported as percentage of gated cells. In panel a, numbers on top of tumor sample bars refer to the difference of percentage between that tumor and the matched normal sample (top line) and to the T/N ratio detected by *tagArray* analysis for the corresponding tags (bottom line).

Flow cytometry was used to measure the binding of three phage-Abs to the primary cells from tumor and normal tissue obtained from pt#78 and pt#80. As reported in Figure 3a, all three phage-Abs differentially bound tumor cells, whereas the matched normal cells and clone HL60-D3-232 retained its binding specificity when formatted as IgG (Mbr-4; Figure 3b). It is worth noting that the binding hierarchy measured by flow cytometry matches that obtained by *tagArray* analysis.

### Identification of target receptors

**Figure 4:**
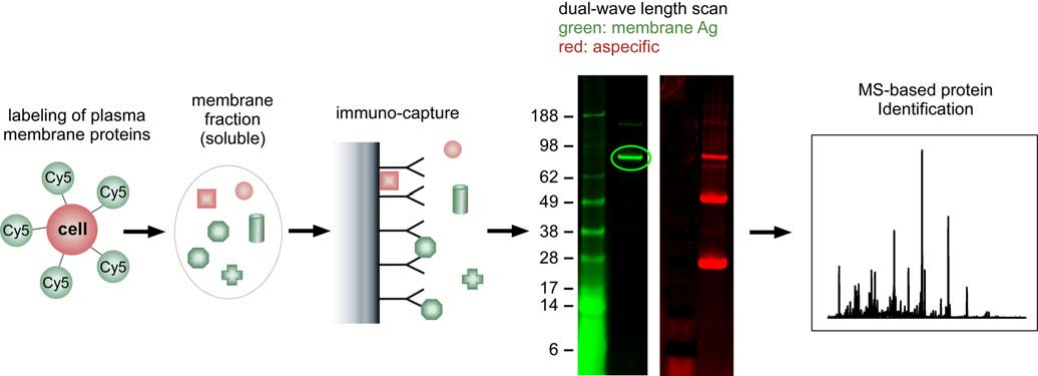
Schematic outline of the biochemical strategy adopted for the identification of the target antigens. Membrane proteins on living cells are selectively labeled with fluorescent dyes (i), then isolated in native conformation and in soluble form by extraction with appropriate buffers (ii). The target antigen is pulled down using the beads-immobilized IgG of interest (iii). Immuno-purified material is analyzed by 1D-PAGE and membrane proteins discriminated from non-specific binders by dual-wavelength scan of the gel (iv). Positive band is excised and processed for MS-based protein identification.

The phage-borne scFv identified as tumor-specific by tagArray analysis were converted into human IgG1 format. Vectors expressing the heavy and the light chains were co-transfected in 293-EBNA cells and the secreted IgGs were affinity purified from the medium and immobilized on beads. Identification of antigens targeted by these monoclonal antibodies (from now on referred to as Mbr-#) followed the strategy illustrated in Figure 4. Membrane proteins on living cells were selectively labeled with fluorescent dye and solubilized in non-denaturing buffer. The proteins immune-purified from this mixture by mAb-coated beads were then analyzed by 1D-PAGE. Bands generated by specific binding of MPs were identified by dual-wavelength scan, excised from the gel and digested for Mass Spectrometry protein identification (see Materials and methods for details). This biochemical approach identified the transferrin receptor (TfRC) as the target of five different mAbs (Mbr-2, 4, 43, 112 and 114). These results were confirmed by detecting a specific binding of the same mAbs to CHO cell transfected with a TfRC expression plasmid (data not shown).

### Biological activity of selected IgGs

We characterized the binding of the mAbs to TfRC by surface plasmon resonance. This analysis revealed K_D_s in the 5–140 nM range with k-off values ranging from 2 to 40 msec^−1^ for the binding of IgGs to the soluble TfRC extra-cellular domain (see Table S3). Apparent K_D_s between 3 and 12 nM on HCT-116 cells were instead obtained using cell-ELISA. A molar excess of the natural ligand transferrin inhibited binding of the five mAbs to TfRC (data not shown).

**Figure 5:**
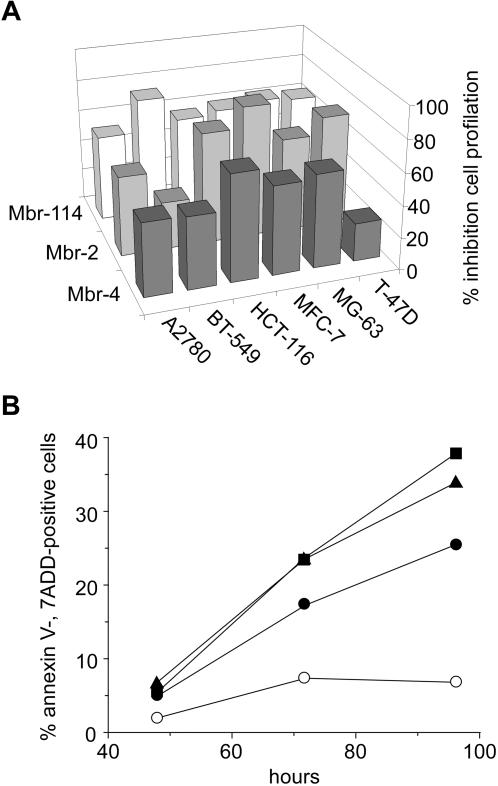
In vitro characterization of anti-TfRC Mbr-4. (a) The indicated cell lines were incubated for 72 hrs at 37°C with 100 µg/mL of Mbr-2, Mbr-4, Mbr-114 or an isotypic control. Cell viability is reported as percentage of the isotopic control treated cells. (b) HCT-116 cells were incubated for 48, 72 and 96 hrs with 0.8 (•), 4 (▪) and 20 (▴) µg/mL of Mbr-4 or with an isotypic control (○). Cell apoptosis is reported as percentage of Annexin V-, 7AAD-positive cells.

Mbr-2, Mbr-4 and Mbr-114 inhibited cell proliferation *in vitro* in 6 cell lines at the concentration of 100 µg/mL (Figure 5a). Mbr-4, the clone exhibiting the highest binding affinity for its target receptor, was further characterized and shown to effectively promote apoptosis of HCT-116 cells at concentrations ranging from 0.8 to 20 µg/L (Figure 5b). Additional experiments indicated that anti-proliferative and pro-apoptotic activities are exerted by competition with transferrin for binding to TfRC (data not shown).

**Figure 6:**
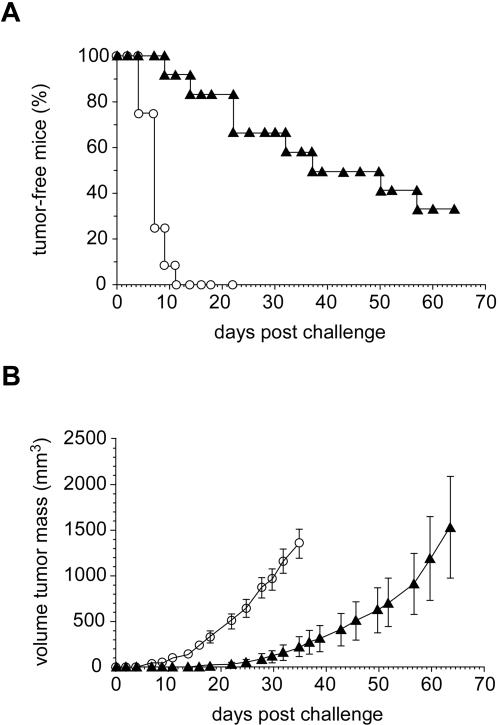
In vivo anti-tumoral activity of anti-TfRC Mbr-4. BALB/c nude mice were challenged with a subcutaneous injection of HCT-116 cells at day = 0. A first group of mice (filled triangles; n = 12) was administered with 10 i.p. injections of 0.5 mg IgG-4 three times a week starting from day = 0. Mice from this group were followed up to day = 64. A second group of mice (empty circles, n = 12) was administered injections of control isotypic IgG according to the same protocol and followed up to day = 35, when mice were sacrificed for ethical reasons. Both groups were monitored for tumor growth expressed as percentage of free mice (panel a) and tumor volume (b). Error bars in panel b indicate standard error of the mean (S.E.M).

Mbr-4 *in vivo* anti-tumor activity was assessed by xenograft experiment (Figure 6a and 6b). HCT-116 cells were injected subcutaneously in BALB/c nude mice, immediately followed by an intra peritoneal injection of Mbr-4 or an isotypic control. Both mAbs were administered 3 times per week for 21 days and the percentage of tumor-free mice and tumor growth monitored: The former dramatically differed between the HCT-116 and BALB/c groups. At day 11 measurable tumors were detected in all control mice (n = 12) whereas only 1 tumor was observed in the group treated with Mbr-4 (n = 12; p<3×10^−5^). On treatment end at day 21, only 2 mice had a tumor in the Mbr-4 treated group. A significant difference between the two groups was detected until day 64 when the experiment was stopped. In line with these results, tumor volume was also lower in the Mbr-4 treated group of mice than in controls.

## Discussion

In the majority of cases the identification of a therapeutic target necessarily precedes the selection of a therapeutic mAb, whereas we demonstrate that this “therapeutic target to mAb” approach can be reversed. Here we describe a strategy which enables the identification of antibodies that bind unknown receptors with a specific expression profile. The first key stage of this strategy is the selection of a relatively low-complexity, “specialized” library containing phage-Abs that bind to MPs (the *Membranome* collection). The second critical element is the development of a technology that enables an efficient differential screening of phage clones, using minimal amounts of biological samples.

We generated the *Membranome* collection by panning a phage-Ab library on cultured cells This process simultaneously selects ligands for several cell surface receptors. The complexity of the library, the display level of the target receptor on the cell surface and the affinity of the interaction between phage-Ab and the target receptor strongly affect this process. All these factors interact in a competitive environment where biological viability of the phage-Ab also plays a major role. Empirically, however, we observed that on average the number of new clones identified by panning on each different cell line remains relatively constant, indicating that we are still far from saturation of the library sequence diversity.

The amino acid sequence of VH3-CDR3, the hypervariable region which in most cases acts as a major binding determinant [6]–[8], is a unique and distinctive feature of each member of the repository. The *Membranome* phage-Ab collection hosts VH-CDR3 sequences ranging from 3 to 25 residues, with the frequency peaking around 10 residues, and analysis of the complete scFv region reveals a broad set of germline families. Together these two findings indicate that a wide variety of binding motifs are present in the collection. We estimate that the current version of the repository (10,000 clones) should target between 500 and 1,000 different antigens, corresponding to 10–20% of the complete *Membranome*. We also attempted to evaluate the diversity of antigens targeted by the phage-Ab repository through two independent approaches (see Text S1), and believe that a conservative figure combining both approaches could be anything between 10 and 20 clones per target. It is worth noting that the 18 target receptors identified so far are membrane proteins with one or two trans-membrane domains and large (>700aa) extra-cellular domains, along with several GPI-anchored membrane proteins.

The tagArray technology efficiently surveys the *Membranome* collection and singles out clones which recognize receptor molecules differentially expressed between two complex biological systems of interest, achieved by coupling phage-display to high throughput oligonucleotide microarray screening. To illustrate a practical application of the *Membranome* phage-Ab collection, we describe the identification of tumor-specific mAbs. An early version of the collection which included 4,000 members was surveyed for clones specifically binding epithelial cells from primary colorectal tumors. This screening revealed that for each patient, several phage-Abs specifically recognized primary cells from tumor versus matched normal tissue although different patients shared only a small fraction of these phage-Abs. A similar finding has been reported for the screening of cDNA expression clones from tumors using autologous or heterologous sera [9].

We focused our attention on the phage-Ab that specifically reacted with tumor cells from two patients. Phage-Abs are good binders and their interaction with the target molecule is efficiently detected through the phage moiety. However, to perform functional assays and to identify the target Ag, we converted the selected phage-Ab into human IgG1. Using biochemical methods combined with mass spectroscopy, TfRC, the main iron transporter which binds and internalizes the iron carrier transferrin, was identified as the target for six of these tumor specific mAbs. Fast-paced tumor replication requires high levels of iron uptake, thus TfRC is often over-expressed in breast, gastric, colon cancer and glioblastoma [10] and has therefore prompted the use of TRC as a target for anti-cancer therapy [11]. Some examples are in a phase II clinical trial, transferrin fused to tetanus toxoid protein showed 30% of responders in glioblastoma patients, leading to the approval of a phase III trial [12]. In another study, a mouse monoclonal was shown to induce apoptosis only in T lymphocytes of acute T-cell leukemia patients, but not in PBMC from healthy donors [13] and in line with this finding, an anti-TfRC IgA showed clinical responses in lymphoma patients. More recently, induction of apoptosis trough TfRC was reported using Gambogic acid on a panel of cell lines [14].

tagArray technology is a versatile tool for profiling protein expression on any cell surface. An increasing number of reports show that, although closely related, transcriptional and proteomic data do not always coincide [15]. Transcriptional profiling has a further drawback of not detecting post-translational modifications or variations in protein stability which also drive important biological processes. Capitalizing on DNA-based technology, the *Membranome* phage-Ab collection offers a complementary approach to standard proteomic analysis by applying unmatched throughput and high sensitivity of microarray technology to protein expression analysis. This method provides a comprehensive multiplexed readout (>20,000 data points) of the expression of receptors displayed on the cell surface, and allows highly sensitive monitoring of their variations. We anticipate that this technology will have a fundamental impact on the identification of disease-associated biomarkers. This same technology has the potential to provide novel information about the biological mechanisms underlying several pathological conditions and, at the same time, deliver useful reagents for validating therapeutic hypotheses. We believe that the strategy and methods described in this paper can be applied to other biological systems such as the secretome, phosphoproteome, acetylome.

## Materials and Methods

### *In Silico* identification of MP-coding genes

All protein coding genes within the NCBI Human Gene database (26,651 genes) were analyzed using the NCBI Gene Ontology (GO) annotation and the *Phobius* transmembrane segment and signal peptide prediction algorithm [16]. A gene was classified as a Membrane Protein (MP) coding gene if either it was annotated as localized in the “Plasma Membrane” according to the Gene Ontology or one of the encoded peptides was predicted to be a membrane protein by the *Phobius* algorithm. Approximately 26% of the human genes were thus classified as MP genes and then subjected to a semi-manual inspection to exclude proteins that are part of intracellular compartments or the nucleus. Genes annotated to be attached to the membrane by a GPI–anchor were manually extracted from databases and added to the list. This left 4,609 MP genes, approximately 19% of all genes (the human *Membranome*) as our target gene population coding for cell surface antigens. In this analysis additional complexity generated by alternative splicing events or post-translational modifications were not taken into account.

### MP genes expression analysis in cell lines

mRNA gene expression data from 25 cell lines (see Table S1) were analyzed for variation of expression levels across the cell lines. Expression levels were inferred using custom designed exon-junction microarrays carrying multiple probes for each transcript as described [17]. To estimate the reliability of absolute mRNA quantification obtained by exon-junction microarrays, 40 genes were independently assayed by TaqMan on the A549 cell line and a good correlation was observed (data not shown). Transcript abundance for about 3,700 MP genes represented on the exon-junction chip was expressed as an interval of 100 to 60,000 arbitrary expression units. “Cell line surface diversity”, here defined as difference in composition of surface membrane proteins, was estimated by the comparative analysis of the highest expressed MP genes. For each cell line, MP genes were ranked according to their expression values and those above an arbitrary ranking threshold (rank < = 200, 300 or 400) were selected as the most abundant membrane proteins. This analysis revealed that a considerable portion of the *Membranome* (about 30%) was expressed at the highest level in only one out of the 25 cell lines, (see Table S1). On the contrary, only a small fraction (around 2–3%) of MP genes showed ubiquitous high expression levels across all cell lines. Comparable results were obtained when, instead of the ranking threshold, an absolute cutoff threshold was applied to expression levels. Individual cell lines contributed to various degrees to the fraction of cell-specific MP genes, with HUVEC and G361 contributing most. In order to obtain a global picture of the cell line surface diversity, a clustering analysis was carried out. All genes expressed above the ranking threshold (rank < = 200, 300 or 400) in at least one cell line were selected and clustered hierarchically (average link) using the euclidean distance measure.

### Phage selection

Human, non-immunized scFv phage-displayed libraries were used as source of binders. These are high complexity libraries (over 10^10^ different clones each) derived from B cells of healthy donors [5]. Each phage-Ab library (about 10^11^ TU) was independently panned on 64 different cell lines (listed in Table S2). In each selection phage libraries were incubated with MPBS (3% powdered non-fat dry milk in PBS solution) for 30 min at room temperature (RT). Human cells used in the selection were detached from the plate using 2.5 mM EDTA in PBS and re-suspended to a final concentration of 1×10^7^ cells/mL. Following centrifugation, cells were re-suspended with pre-adsorbed phage-Abs solution and incubated for 1 hour at RT. After extensive washing with PBS, cell-bound phage were centrifuged 5 min at 2 k rpm and re-suspended in 800 µL of trypsin solution (1 µg/ml in sodium phosphate). *E. Coli* TG1 cells (New England Biolabs, Beverly, MA) were infected with eluted phage and plated on 2XTY agar containing 2% glucose and 100 µg/mL ampicillin (2XTYAG). Phage rescue and amplification was carried out as described [18]. The selected phage were panned again on the same cell line. A variable number of clones (in the range 100–1,000) were randomly chosen form the pool of selected phage and tested by phage ELISA for their ability to bind the same cell line.

### Whole-cell phage-ELISA

Cells were seeded overnight in 96 well plates at the concentration of 4×10^4^ cells in 100 µL of propagation medium per well. Following blocking with MPBS, phage supernatants were added to each well and incubated for 1 hour at RT. After washing with PBS, binding of phage antibodies was revealed with HRP-conjugated anti-M13 antibody (Amersham Biosciences, Pittsburg, PA) in MPBS, using tetramethylbenzidine (Sigma Aldrich, Chicago, IL) as substrate. Phage-Abs whose binding to the target cell line measured by A_450nm_-A_620nm_ was at least three fold higher than that observed with an unrelated phage and higher than 0.3 units were defined as positive.

### tagArray repertoire

We identified 12 sequences sharing the following properties: i) 7 nucleotides long; ii) G2W5 base composition; iii) do not contain G-C palindromes; iv) do not contain self complementary sequences; v) have the same melting temperature, which is 10°C higher as compared to each of the other 11 sequences. By combining 4 of these building blocks we generated a repertoire of 20,736 (12^4^) sequences of 28 nucleotides. Each member of this repertoire has a 10°C difference or higher in Tm with any other member of the repertoire.

### Cloning the tag repertoire

Phagemid pCANTAB5 DNA was linearized by SapI restriction and a synthetic dsDNA fragment with compatible cohesive ends was inserted (AGCCTTAATTAATACGACTCACTATAGAGGCCTGGTACCCGGGTCGACTGTGTGTCT). The resulting vector pCB5-PacI/XmaI has unique PacI and XmaI restriction sites (underlined in the sequence above).

An oligonucleotide was synthesized by “mix and split” procedure which contained the complete (G2W5)_4_ sequence repertoire flanked by constant regions (tagrep; CCTTAATTAATACGACTCACTATAG(G2W5)_4_ CCCGGGGG). An oligonucleotide with sequence GGCCCGGG was annealed to tagrep template and extended with DNA polymerase Klenov fragment. The double-stranded DNA product thus generated was digested with PacI and XmaI restriction enzymes and cloned into the corresponding sites of pCB5-PacI/XmaI (pCB5-(G2W5)_4_ library). By transformation in *E.coli* TG1 cells a number of colonies much higher than the library complexity was obtained.

### Tagging the 4k Mbr collection

The phagemid DNA derived from the 4k *Membranome* phage-Ab collection was digested with SfiI and NotI restriction enzymes. The 800bp fragment including the scFv sequences was purified and cloned into the corresponding sites of the pCB5-(G2W5)_4_ library. Upon transformation in *E.coli,* TG1 cells of about 10,000 colonies were collected. Indeed, a computer-aided simulation indicated 2.5 times the scFv complexity as the best compromise between library complexity and the association of multiple tags to the same scFv sequence. A similar protocol was adopted to tag the 10k *Membranome* phage-Ab collection.

### Epithelial cell immunoaffinity purification

Tumor epithelial cells were purified using a protocol derived from Buckhaults et al [19]. In brief, fresh dissected tissue was digested with collagenase and DNase at 37°C in DMEM+ (DMEM, 1% FCS, 20 mM HEPES, Penicillin-Streptomycin). The resulting material was filtered through a nylon mesh to obtain single cell suspensions. After red blood cells lysis, cells were immediately used for panning or re-suspended in 10% DMSO/FBS and stored in liquid N_2_. In either case, before use, Ep-CAM positive epithelial cells were selected using autoMACS separator (Miltenyi Biotec, Bergisch Gladbach, Germany) according to manufacturer's instruction, and tested for their via1bility using Guava Personal Cytometer (Guava Technologies Inc., Hayward, CA).

### tagArray screening

The tagged 4 k Mbr phage-Ab library was panned on primary human epithelial cells derived from tumor or matched normal samples as described above. Usually 10^7^ cells were incubated with 10^11^ phage. Following extensive washing, cell-bound phage were eluted and used to infect TG1 cells. The Ampicillin-resistant bacterial colonies were collected and phagemid DNA purified by DNA purification kit (Qiagen GmbH, Hilden, Germany). Tag sequences were amplified by PCR. Forward primer was 5′-labelled with Cy5 or Cy3 dyes. Reaction mix included 30 ng phagemid DNA, 5 µM labeled forward and reverse primers, 200 uM dNTPs and 2.5 units Taq polymerase (Promega, Madison, WI) in a final 50 µL volume. Nine explicitly tagged-phagemids with different relative ratios and absolute amounts were added to the reaction. These “spike in” probes monitored whether the amplification process maintained the original relative frequencies of the clones in the amplified population. The amplification product was first purified from salts, free primers and dNTPs by a commercially available removal kit (Qiagen GmbH, Hilden, Germany). An aliquot was then run onto an agarose gel and the Cy5- or Cy3-labeled fragment was quantified by a fluorescence scanner (Typhoon; Perkin Elmer, Waltham, MA).

The Hybridization mix was assembled in a 490 µL final volume containing 3.5 mM Cy5- and Cy-3 labeled probes, 0.5 µM blocker and reverse primer. The latter perfectly hybridizes to the phagemid sequences flanking the tag, thus allowing the tag to interact with complementary tag sequences immobilized on a custom designed microArray chip (Agilent Technologies, Palo Alto, CA). The reaction mix was incubated at 99.9°C for 2 minutes and then snap-cooled by transferring back in ice for 1 min. The mix was then applied onto *tagArray*_44 k chip and incubated at 64°C for 15 hours in a rotating chamber. The slide was then washed at RT in 6xSSPEL (6× SSPE, 0.005% N-Lauroylsarcosine), 0.06xSSPEL, rinsed in stabilization and drying solution (Agilent Technologies, Palo Alto, CA) and analyzed with Agilent scanner. Images were acquired at 10μm resolution using an Agilent scanner, with the XDR option enabled (eXtended Dynamic Range: for each slide two images were generated with photomultiplier tube voltages of 100 and 10, respectively). Images were then processed using the Feature Extraction software (v 9.1, Agilent Technologies, Palo Alto, CA) generating the net signal for each channel (Cy3 and Cy5) and p-values after background and dye-bias correction. Results reported were obtained by combining data from two independent fluor reversal experiments.

### Rescue scFv associated to selected tags

For each tag sequence we generated a primer including the two 5′ blocks (X_1_X_2_) preceded by the adjacent 5′-CGACTCACTATAG-3′ constant region (primer dynoX_1_X_2_) and a primer encompassing the 4 blocks (primer X_1_X_2_X_3_X_4_). A reverse primer complementary to the phagemid region downstream of the scFv was used in conjunction with the dynoX_1_X_2_primer in a PCR reaction using the T phagemid DNA population as template. The product of this first amplification was diluted and re-amplified using the primer X_1_X_2_X_3_X_4_ and the reverse primer. The PCR products obtained were sequenced and the corresponding 10k *Membranome* phage-Ab clones identified.

### scFv to IgG1 conversion

The scFv VH and VL sequences were introduced into a mammalian expression plasmid to produce the IgG1 heavy or light chain by using the Gateway™ technology (Invitrogen, Carlsbad, CA). In our hands, the frequency of recombinant clones was higher than 90% for both VH and VL insertion, close to the efficiency reported for different vector systems based on Gateway technology. The two plasmids secreting the IgG1 light and heavy chains were co-transfected into 293-EBNA cells. Cell culture medium was harvested every 3 days, samples collected together and purified on protein A affinity column.

### Target antigen identification

A-549, HCT-116 and MCF7 cells were grown at 70–80% confluence and cell surface proteins labeled with Cy5 fluorescent mono-reactive dye (Amersham, Uppsala, Sweden). About 20% of the cells were washed with PBS and incubated for 30 minutes at 4C° in the presence of Cy5 dye (usually 1 vial of Cy5 dye/10^8^ cells). The Cy5 solution was removed and the cells washed with PBS. Cy5-labeled and unlabeled cells were then incubated for 30 min at 4%C in non-denaturing lysis buffer (50 mM Tris-HCl pH 7.5, 150 mM NaCl, 1% Triton-X100), complemented with Protease Inhibitors cocktail (Amersham, Uppsala, Sweden) at a concentration of 10^6^ cells/mL. The supernatant of Cy5-labeled and unlabeled cells was then recovered, pooled and cleared by centrifugation. The native membrane protein preparations (NMPs) thus generated were immune-precipitated by incubation with a mAb covalently bound to CNBr-activated sepharose beads (Amersham, Uppsala, Sweden) at +4°C for 2hrs on a rotating wheel. The beads were then washed with lysis buffer and the immune-precipitated proteins were eluted in reducing loading buffer and analyzed by dual-wavelength 1D-PAGE using a Typhoon 9410 (Amersham, Uppsala, Sweden). Protein identification was performed essentially according to Shevchenko *et al.* [20]. Briefly, the protein bands specific for membrane proteins were excised and digested *in-gel* with modified porcine trypsin (Promega, Madison,WI). Tryptic peptides were extracted from the gel pieces, dried down, resuspended in 0.1% Trifluoracetic Acid and desalted by µC_18_ ZipTip (Millipore, Bedford, MA). Peptides were then analyzed either by Peptide Mass Fingerprint experiments (MALDI-TOF-MS) using a Voyager DE sSTR (Applied Biosystems, Foster City, CA) and the MASCOT software package or by capillary LC-ESI-IT-MS/MS using a LCQ Deca XP Plus (Thermo, Waltham, MA) equipped with a micro-electrospray source connected to a in-house packed C18 column (100 mm × 0.10 mm) and the TurboSequest software (Xcorr values >2.5).

### Surface Plasmon Resonance

Sensorgrams were recorded on a Biacore 3000 instrument operating at 25°C with CM5 chips (Biacore AB, Uppsala, Sweden). The antibodies were immobilized using the unmodified amine-coupling chemistry procedure suggested by Biacore. Regeneration conditions were established for each antibody according to the return of the baseline to the initial values. Double referencing was used by subtracting the sensogram resulting from an injection of running buffer. Mass transport influence was minimized combining flow rate and low amount of mAbs immobilized (ranging within 500–2000 RU). The titrations to determine the kinetic constants of complex formation were designed with 10 different analyte concentrations, plus one duplicate concentration. All the titrations were performed in duplicates with the kinject mode, using a flow rate of 50 µL/min and a constant time (3 min). The data were analyzed with the Bia-Evaluation 4.1 software using the Langmuir 1:1 binding model, and the results are given as averages of the duplicates. When feasible, the titrations were also performed at equilibrium. In this case the flow rate used was 10 µL/min, with a contact time of around 6 min. The equilibrium data were analyzed using the equilibrium model within the Bia Evaluation software.

### Flow cytometry

PEG-precipitated phage-Abs were re-suspended in PBBS (2% BSA in PBS) and incubated for 30 min at RT. Primary human cells obtained from tumor or normal tissue were incubated with phage-Ab solution 60min at 4°C (2×10^5^ cells with pre-blocked phage in 100 µL). After washing with PBBS, cells were incubated with biotin-conjugated anti-M13 phage monoclonal antibody (Progen Biotechnik, Heidelber, Germany) for 30 min at +4°C. then washed and incubated with streptavidin-APC (Molecular Probes/Invitrogen, Carlsbad, CA) or with anti- Ep-CAM FITC Mab (Miltenyi Biotec, Bergisch Gladbach, Germany) 30 min at 4°C. Finally, cells were washed and fixed with 1% PAF in PBS. mAbs were labeled by incubation with Zenon Human IgG APC labeling kit (Molecular Probes/Invitrogen, Carlsbad, CA) according to manufacturer's guidelines. Target cells were then incubated with pre-labeled mAb solution for 30 min at RT (2×10^5^ cells in 100 µL of PBBS for 30 min). The same test was performed by using the anti Ep-CAM FITC MAb (Miltenyi Biotec, Bergisch Gladbach, Germany). In both cases cells were then washed and fixed with 1% paraformaldehyde in PBS.

### Mice

Mice were bred under specific pathogen-free conditions by Charles River Breeding Laboratories (Calco, Como, Italy). In all manoeuvres, mice were treated in accordance with European guidelines. In particular, at the time of injection mice were fully anesthetized with ketamine (Merial Italia, Milano, Italy) at 100 mg/kg of body weight and xylazine (BIO 98; Bologna, Italy) at 5.2 mg/kg.

### Cell viability and apoptosis assays

HCT-116 cells were cultured in McCoy's supplemented with 10% fetal calf serum (FCS) in a humidified atmosphere with 5% CO_2_ at 37°C. For cell viability assays, cells were seeded in 96-well plates (10^3^ cells/well) and incubated for 72 hrs with 100 µg/mL of Mbr-4 or an isotypic IgG used as negative control. Cell viability was then measured using a fluorometric assay according to the manufacturer's instructions (Cell Titer Blue assay; Promega, Madison, WI) and expressed as percentage of the value measured by cells treated with the negative control. For cell apoptosis assays, cells were seeded in 24-well plated (5×10^3^ cells/well) and incubated for 48, 72 and 96 hrs with 0.8, 4 and 20 µg/mL, respectively, of mAb-4 or an isotypic IgG as negative control. As positive control 500nM apoptosis inducer Staurosporine was also tested. Cell apoptosis was monitored by measuring Annexin V and 7-aminoactinomycin (7-AAD) expression using a Guava Personal Cytometer (Guava Technologies Inc., Hayward, CA) and expressed as percentage of the Annexin V-, AAD-positive cells. No variation in the percentage of necrotic cells was detected. Data were analyzed with one-way ANOVA (p<0.05).

### In vivo experiments

BALB/c nude mice were challenged with a subcutaneous injection of 4×10^6^ HCT-116 cells (day 0). The same animals were injected with 0.5 mg of Mbr-4 or a control isotypic IgG at day 0, 2, 4, 7, 9, 11, 14, 16, 18, and 21 (12 animals per group). Mice were inspected three times a week and tumor masses measured with calipers in two perpendicular diameters. Growth was monitored until a tumor exceeded an average diameter of 10mm, at which time mice were euthanized for humane reasons. Data reported are representative of two independent experiments.

## Supporting Information

Table S1 (0.02 MB XLS)

Table S2 (0.02 MB XLS)

Table S3 (0.01 MB XLS)

Text S1 (0.02 MB DOC)
