# Supplementary material for: Differential Screening of Phage-Ab Libraries by Oligonucleotide Microarray Technology
Source: PLoS One. 2008 Jan 30;3(1):e1508. doi: 10.1371/journal.pone.0001508 (PMC2204054; doi:10.1371/journal.pone.0001508)
Supplement: Text S1 — (0.02 MB DOC) [file pone.0001508.s004.doc]

**Text S1**

**Diversity of antigens recognized by the *Membranome* phage-Ab collection**

The diversity of antigens targeted by the phage-Ab repository was evaluated through two independent approaches.

1. 10k Membranome collection was assayed for binding to five different cell-surface receptors. According to this analysis, 151 clones (about 1.5% of the population) bound one of the five target receptors, with the number of phage-Ab targeting the same antigen ranged from 6 to 54, with an average of 30±20. This rough estimate, however, is biased by the choice of the target antigens. These have been identified as targets of antibodies obtained during the first round of selections where more abundant and antigenic receptors are expected to drive the selection of ligands.
2. 124 clones were randomly chosen from the Membranome collection, converted into IgGs and processed according to the biochemical strategy described. Sixty-two mAbs (50%) did immuno-precipitate a detectable amount of target antigen. In 38 cases the cognate antigens were unambiguously identified and accounted for 18 different proteins. These data indicate an average ratio of 2.1 clones binding the same target (standard deviation lower than 1.8).
